# Association Between Anti-TNF Therapy for Rheumatoid Arthritis and Hypertension: A Meta-Analysis of Randomized Controlled Trials

**DOI:** 10.1097/MD.0000000000000731

**Published:** 2015-04-10

**Authors:** Qingwei Zhao, Dongsheng Hong, Yi Zhang, Yanlei Sang, Zhihai Yang, Xingguo Zhang

**Affiliations:** From the Department of Pharmacy (QZ, DH, YZ, YS, ZY, XZ), the First Affiliated Hospital of College of Medicine, Zhejiang University; and College of Pharmaceutical Science (XZ), Zhejiang Chinese Medical University, Hangzhou, P.R. China.

## Abstract

Tumor necrosis factor (TNF) is an important and pleiotropic cytokine which is also involved in the pathogenesis of inflammation in rheumatoid arthritis (RA), and RA treated with anti-TNF agents with a subsequent increase in hypertension risk is also observed in clinical trials. However, it is confusing that to what extent treatment with anti-TNF agents for RA might be associated with increasing risk of hypertension.

The aim of this study was to investigate the overall incidence and risk of hypertension in RA patients who receive anti-TNF agents.

The databases of Embase, PubMed, the Cochrane Library, and clinical trial registration Web site were searched for relevant trials. Statistical analyses were conducted to calculate the overall incidence, odds ratios, and 95% confidence intervals (CI) by using either random-effects or fixed-effect models according to the heterogeneity of the included studies.

A total of 6321 subjects with RA from 11 randomized clinical trials (RCTs) were included in the meta-analysis. The overall incidence of hypertension associated with anti-TNF agent was 3.25% (95% CI: 1.51%–6.89%). The use of anti-TNF agent significantly increased the risk of developing hypertension (OR = 1.8896, 95% CI: 1.35–2.65). Sensitivity analysis showed that the OR between anti-TNF therapy and controls is not significantly influenced by omitting any single study. No evidence of publication bias was observed.

Anti-TNF therapy is associated with a significantly increased risk of developing hypertension in patients with RA. Physicians should be aware of this risk and provide continuing monitoring in patients receiving these therapies.

## INTRODUCTION

In the past decade, the treatment of anti–tumor necrosis factor (anti-TNF) agents in patients with rheumatoid arthritis (RA) has been demonstrated to be efficacious in preventing progress of structural damage and functional deterioration,^1–3^ and RA treated with anti-TNF agents with a subsequent increase in hypertension risk is also observed in clinical trials.^4,5^ Up to present, it is confusing that to what extent treatment with anti-TNF agents for RA might be associated with increasing risk of hypertension.^6–9^ This confusion is based on the difficulties of interpretation and analysis of few adverse event data derived from randomized controlled trials (RCTs), and hypertension is a clinical feature with poor prognosis since its pathophysiology has not yet to be clarified and therapeutic options are limited. It is important to understand the incidence of hypertension.

TNF is an important and pleiotropic cytokine which is also involved in the pathogenesis of inflammation in RA.^10^ Basic science research showed that hypertension overexpressed TNF which played a vital role in the hypertension's occurrence and development,^11^ and chronic infusion of TNF is adequate to mimic some aspects of heart failure (HF), including progressive ventricular dysfunction and cardiomyocyte hypertrophy; some of which can be reversed by treatment of anti-TNF agents.^12,13^

According to FDA, anti-TNF agents licensed for clinical use in RA including infliximab, etanercept, adalimumab, certolizumab pegol, and golimumab.^14–18^ Lots of clinical trials reported that patients in the duration of anti-TNF agent treatment developed hypertension,^19–23^ and hypertension should be seriously considered as possible adverse effects of anti-TNF agents. RCTs in patients with RA have been inconsistent, with some showing significant and others nonsignificant association between hypertension and anti-TNF therapy.^19–23^ In addition, RCTs have been too brief or too small to accumulate enough hypertension events, and data concerning hypertension with anti-TNF agents used in different clinical trials have not been evaluated, the association between anti-TNF agent therapy for RA and hypertension is uncertain. Therefore, we conducted this meta-analysis (MA) to assess the incidence and risk of hypertension of anti-TNF agent in RA patients.

## METHODS

### Search Strategy and Study Selection

We searched Embase (dates from 1974 to 2014), PubMed (dates from 1967 to 2014), and the Cochrane Library electronic databases. Specifically, we used the following search terms treated as Mesh terms or free text: “Rheumatoid arthritis,” “Arthritis, Rheumatoid”; “Infliximab,” or “Etanercept,” “Adalimumab,” “Certolizumab pegol,” “golimumab,” “D2E7,” “cA2,” “CDP870,” “TNFR-Fc,” “CNTO148”; and “Randomized controlled trials,” “Clinical trials,” “Controlled clinical trials,” “Clinical trial as topic,” or “Randomized controlled trial as topic.” Additionally, we also searched the clinical trial registration Web site (ClinicalTrials.gov) to obtain information on the registered clinical trials. Detailed search strategies are shown as Supplemental Content. This study is an MA and not involves subjects, ethical approval was not required.

Study selection was conducted according to the Preferred Reporting Items for Systematic Reviews and Meta-Analyses (PRISMA) Statement.^24^ Clinical trials that reported the occurrence of hypertension with anti-TNF antibody use in RA patients were eligible for inclusion, and other inclusion criteria included the diagnosis of RA based on American College of Rheumatology criteria^25^; participants assigned to treatment with an anti-TNF antibody including infliximab, etanercept, adalimumab, certolizumab pegol, and golimumab (alone or in combination at any dosage or frequency); and treatment with anti-TNF antibody for a minimum duration of 12 weeks.^26^ This duration was chosen based on the fact that a study of this duration could provide relevant information on hypertension.

### Data Extraction and Quality Assessment

Hypertension was extracted from the safety profile in each Trial. Two investigators (Z. Q. W. and H. D. S.) extracted date independently, and studies were retrieved for further consideration if judged pertinent by 1 or 2 reviewers. Discrepancies were identified and resolved by consensus or, as needed, by a third investigator (Z. X. G.) and confirmed by consensus. When there were multiple reports from the same trial, the most complete and/or most recently reported data were chosen.

For each study, the following information was extracted: first author's name, year of publication, treatment arm, duration of trial, mean age, mean duration of diseases, number of patients in the treatment and control groups, adverse outcomes (hypertension). All of the RCTs included in this review had their quality assessed using the Jadad criteria.^27^ Scores ranged from 0 to 5 with a high score indicating a high-quality study.

### Data Analysis

MAs were conducted based on the Cochrane handbook.^28^ The principal summary measures were incidence, odds ratio (OR), and corresponding 95% CI. For the calculation of incidence, the number of patients with hypertension in the anti-TNF antibody group and the total number of patients receiving anti-TNF antibody were extracted, and the proportion of patients with hypertension and the 95% CI were derived in each study. The OR of hypertension was calculated only with those assigned to the control group in the same trial. We used the Peto method to calculate the OR and the 95% CI because this method provides the best confidence interval coverage and it was more powerful and relatively less biased when dealing with low event rates.^29^ Heterogeneity was assessed by using the Q statistic and I^2^ tests among clinical trials.^30,31^ Heterogeneity was considered statistically significant when *P* < 0.1 or I^2^ > 40%.^28^ If heterogeneity existed, the data were analyzed using a random-effects model; if heterogeneity did not exist, a fixed-effects model was used. To assess the stability of results, sensitivity analysis was carried out by sequential omission of individual studies. A statistical test with a *P* value less than 0.05 was considered significant. The presence of publication bias was evaluated by using the funnel plot, and Begg and Egger tests.^32,33^

All data analyses were performed by using Stata software, version 12.0 (Stata Corporation, College Station, TX) and R software, version 3.0.3 (the R foundation for statistical computing, http://www.r-project.org).

## RESULTS

### Search Results and Trial Characteristics

**FIGURE 1:**
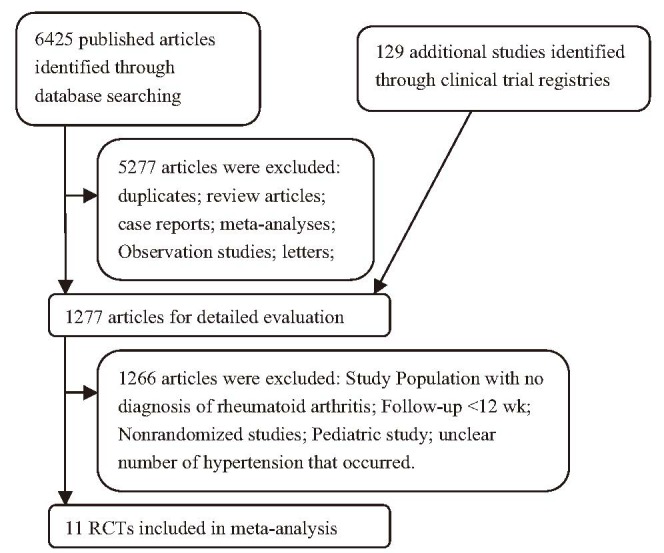
Flow chart demonstrating the process of study selection.

A total of 6425 articles and 129 clinical trials were identified initially through our search. After reviewing each study, 6543 studies were excluded (Figure 1). The remaining 11 studies, with 6321 subjects, which met our inclusion criteria, were included in our analyses.^4,5,19–23,34–37^ The basic characteristics of the trials included in the MA are summarized in Table 1. The quality of the 11 clinical trials was high: 6 of them had Jadad scores of 5,^5,19–21,35,36^ which described the methods of randomization and blinding appropriately and provided the number of patients who withdrew and dropped from the trials. Four clinical trials had Jadad scores of 4.^4,22,34,37^ This lower score was due to the fact that the researchers did not describe the methods of randomization or blinding appropriately. One study had Jadad score of 3.^23^ We performed this MA in accordance with the guidelines of the PRISMA Statement (see Guidelines Checklist).

**TABLE 1:**
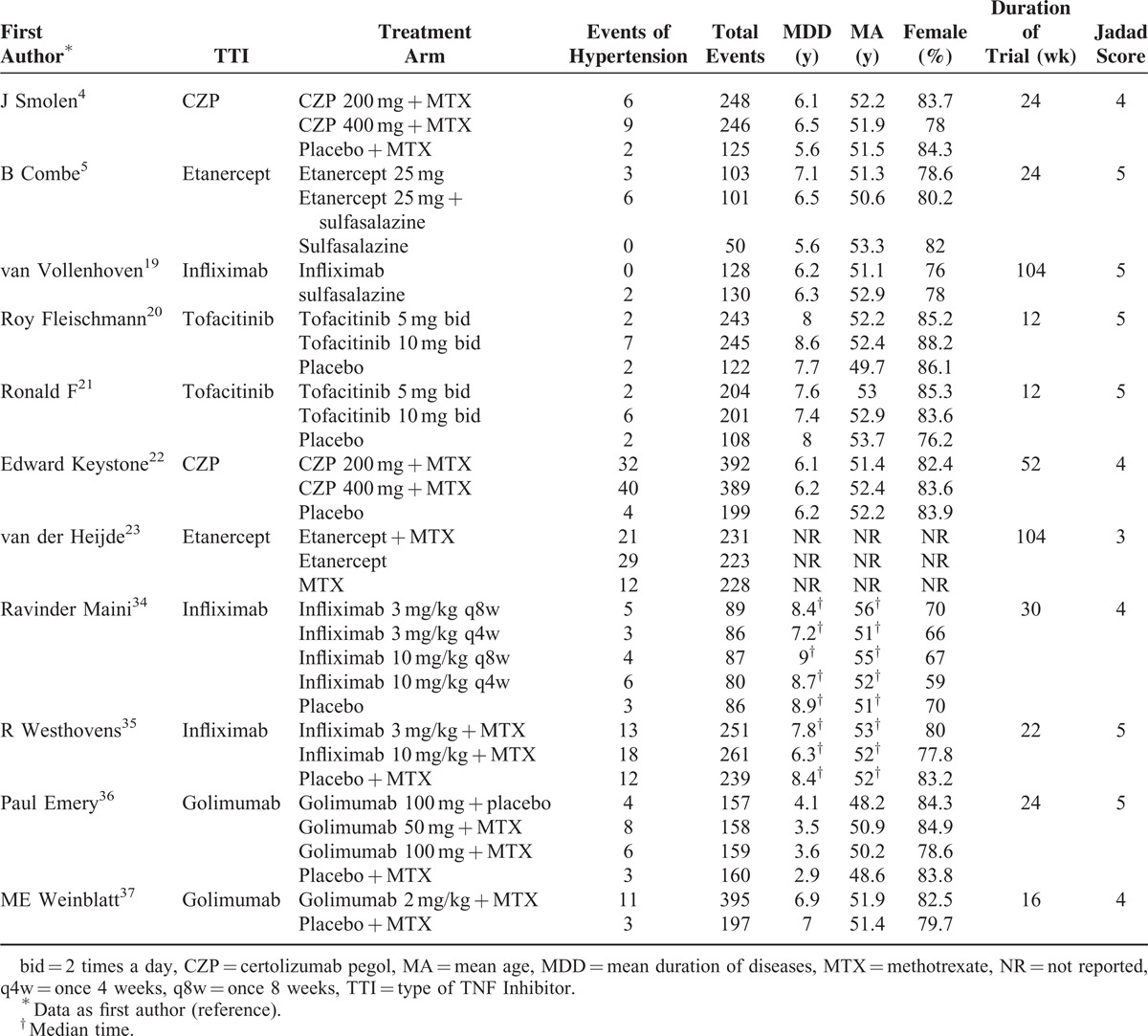
Baseline Patient Characteristics (n = 6321)

### Incidence of Hypertension

**FIGURE 2:**
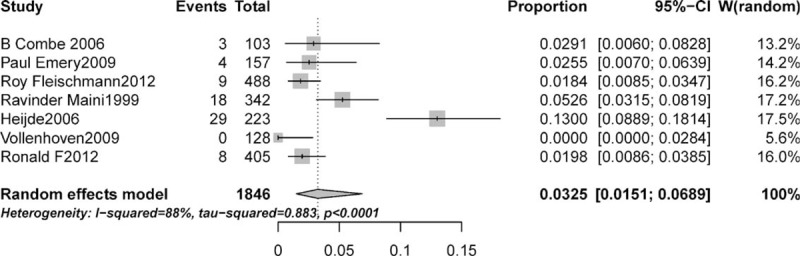
Forest plot for meta-analysis of incidence of hypertension in patients with rheumatoid arthritis.

A total of 1846 patients who were treated with anti-TNF agent monotherapy were available for analysis.^5,19–21,23,34,36^ There were 71 total hypertension events among these patients. The incidence of hypertension ranged from 0% to 13%, and the highest incidence occurred in the trials of patients treated with etanercept during a 2-year period.^23^ Based on data from included trials, the overall incidence of hypertension was 3.25% (95% CI: 1.51%–6.89%; Figure 2) according to the random-effects model (*P* < 0.001; I^2^ = 90%).

### Relative Risk of Hypertension

**FIGURE 3:**
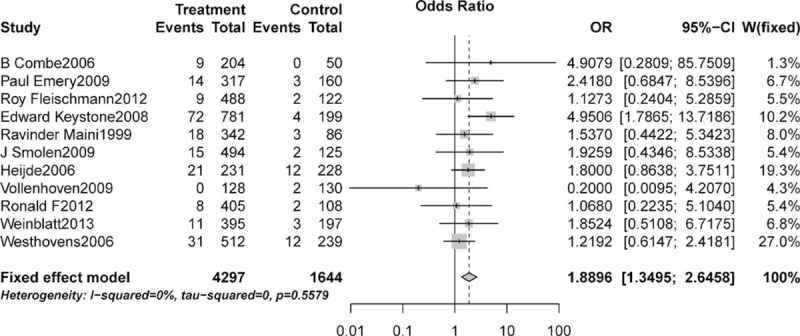
Relative risk of associated hypertension with anti-TNF agent versus controls.

**FIGURE 4:**
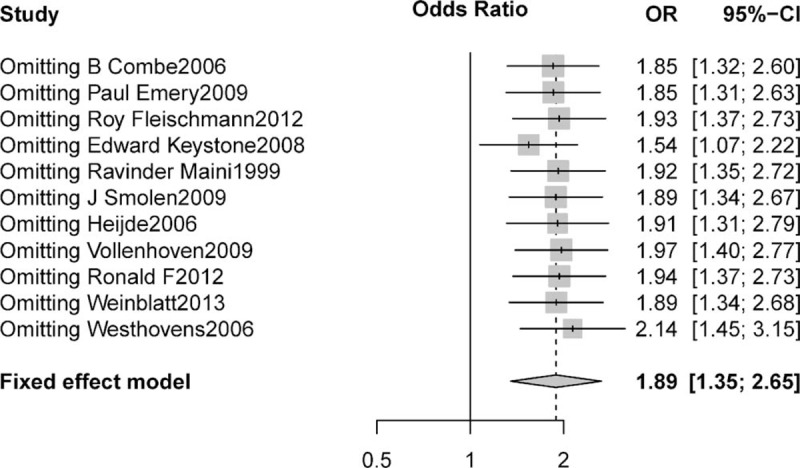
Sensitivity analysis of associated hypertension with anti-TNF agent versus controls.

To investigate the specific contribution of anti-TNF antibody to the incidence of hypertension and exclude the influence of confounding factors, such as food, the disease itself, and the history of other therapeutic interventions, we determined the OR of hypertension between anti-TNF antibody and control groups. The pooled OR of hypertension demonstrated that anti-TNF antibody significantly increased the risk of hypertension comparing with control with an OR of 1.8896 (95% CI: 1.35–2.65; *P* value = 0.0002, Figure 3) according to a fixed-effects model (I^2^ = 0%, *P* = 0.5579).^4,5,19–23,34–37^ To validate the relative risk of hypertension between anti-TNF antibody and control groups, we also performed sensitivity analysis to examine the stability and reliability of pooled OR by sequential omission of individual studies. The results indicated that the significance estimate of pooled OR was not significantly influenced by omitting any single study (Figure 4).

### Subgroup Analysis for OR of Hypertension

The OR of hypertension might be different among duration of trial or type of different TNF inhibitor; we thus performed subgroup analysis according to duration of trial and TNF inhibitors type. The significantly increased risk of hypertension with TNF inhibitor was observed in patients whose treatment time was more than half of a year (OR = 2.35, 95% CI: 1.42–3.89, *P* value = 0.0009, Table 2),^4,5,20,21,35–37^ and an increased risk of hypertension with TNF inhibitor but nonsignificantly was observed in patients whose treatment time was less than half of a year (OR = 1.56, 95% CI: 0.99–2.46, *P* value = 0.055, Table 2).^19,22,23,34^ When stratified by TNF inhibitors type, a significantly increased risk of hypertension was observed in patients treated with certolizumab pegol (OR = 3.62, 95% CI: 1.50–8.73, *P* value = 0.0002, Table 2),^4,22^ and a nonsignificantly increased risk of hypertension was observed in patients treated with etanercept (OR = 1.92, 95% CI: 0.94–3.90, *P* value = 0.053, Table 2),^5,23^ tofacitinib (OR = 1.10, 95% CI: 0.37–3.30, *P* value = 0.867, Table 2),^20,21^ infliximab (OR = 1.20, 95% CI: 0.67–2.16, *P* value = 0.588, Table 2),^19,34,35^ and golimumab^36,37^ (OR = 2.12, 95% CI: 0.86–5.23, *P* value = 0.099, Table 2).

**TABLE 2:**
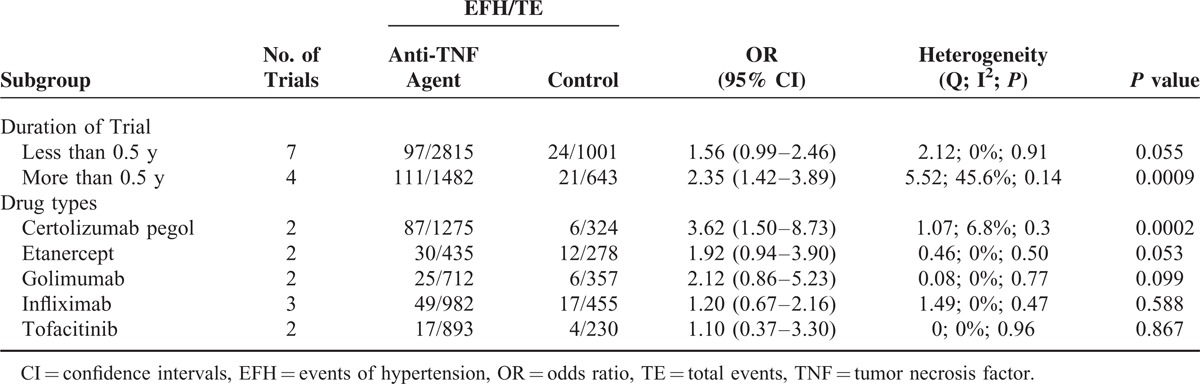
Odds Ratio of Hypertension According to Prespecified Subgroups

### Publication Bias

**FIGURE 5:**
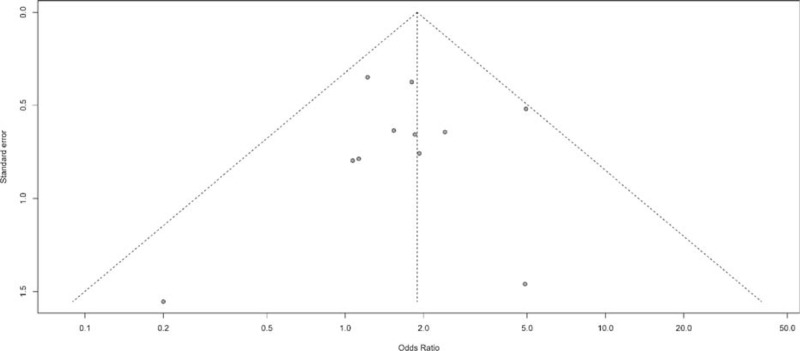
Funnel plot standard error by odds ratio for diarrhea.

No evidence of publication bias for the OR of hypertension was found in our MA analysis by a funnel plot (Figure 5), Egger test (*P* value = 0.903 > 0.05, 95% CI: −1.64, 1.47), or Begg test (Z = 0.47 < 1.96, *P* = 0.64 > 0.05).

## DISCUSSION

Hypertension is a common and one of the most important risk factors for cardiovascular disease during anti-TNF therapy, and concerns have arisen about the risk of hypertension with the use of these agents.^20,38^ Several cohort researches have shown that inflammation cytokines are associated with an increased risk of hypertension.^39–41^ TNF levels have been shown a positive association with hypertension in some studies,^42^ but others have not.^43^ Data concerning hypertension with anti-TNF agent use in different RCTs were also inconsistent.^19,20,37^ Therefore, we conducted this MA to assess the risk and incidence of hypertension of anti-TNF agent in RA patients.

To the best of our knowledge, this is the first MA to investigate the risk of hypertension associated with anti-TNF agent in RA patients. Our analysis, based on 6321 patients from 11 clinical trials, demonstrates that the pooled incidence of anti-TNF agents associated hypertension is 3.25% (95% CI: 1.51%–6.89%). Additionally, we also find that the use of anti-TNF agent is associated with a significantly increased risk of hypertension when compared to controls. This association appears to be time dependent for hypertension and is derived from high-quality randomized trials. Sensitivity analysis showed that the OR between anti-TNF therapy and controls is not significantly influenced by omitting any single study. Based on our results, we could conclude that while anti-TNF agent is associated with an increased risk of developing hypertension in patients with RA, the absolute incidence and risk of hypertension appears low, and the use of anti-TNF agent should be considered in the context of overall survival (OS) benefits.

The significant effectiveness of anti-TNF agent redefined treatment for RA, most importantly because of the ability of these drugs to improve disease activity and prevent a mutilation course in patients who fail to respond to traditional therapy of DMARD. Meanwhile, our analysis provides to the findings that challenge the security profile of anti-TNF therapy. The detected increase of hypertension risk has to be explained according to the notable effectiveness of anti-TNF treatment in RA patients and the short of replacement therapy in cases with serious disease activity adiaphorous to conventional DMARD therapy. The increase in quality of life, gain in mobility, diminution of joint destruction, even in patients with RA who are irresponsive to therapy prior to the introduction of anti-TNF treatment, must be taken into account when considering benefits in individual patients or treatment of risk. The powerful anti-inflammatory impacts of anti-TNF therapy may have a beneficial effect on the OS of patients with RA if they could improve cardiovascular function and reduce disease activity, which are the main cause of death in patients with RA.^44,45^

Our MAs showed an accumulation of hypertension with longer study duration. The significantly increased risk of hypertension with anti-TNF therapy was observed in patients whose treatment time was more than half of a year (OR = 2.35, 95% CI: 1.42–3.89, *P* value = 0.0009),^4,5,20,21,35–37^ and an increased risk of hypertension with TNF inhibitor but nonsignificantly was observed in patients whose treatment time was less than half of a year (OR = 1.56, 95% CI: 0.99–2.46, *P* value = 0.055).^19,22,23,34^ This could be explained that RA patients with prolonged exposure to the anti-TNF agents might result in clustering of events of hypertension, and anti-TNF agents could accelerate of preexisting subclinical hypertension rather than remission. Accordingly, thorough screening for subclinical hypertension of patients being considered for anti-TNF agent therapy and continuous monitoring may represent a therapeutic strategy to improve the safety of anti-TNF treatment that deserves further evaluation.

We also carried out a subgroup analysis according to anti-TNF agents. Our result showed that the risk and incidence of hypertension among different anti-TNF agents are discrepant. A significantly increased risk of hypertension was observed in patients treated with certolizumab pegol (*P* value = 0.0002),^4,22^ and a nonsignificantly increased risk of hypertension was observed in patients treated with etanercept (*P* value = 0.053),^5,23^ tofacitinib (*P* value = 0.867),^20,21^ infliximab (*P* value = 0.588),^19,34,35^ and golimumab^36,37^ (*P* value = 0.099). The fundamental differences in their molecular structures may explain differences in hypertension risk. Among patients receiving certolizumab pegol were significantly associated with increased risk of hypertension; this result is consistent with the previous study.^46^

MA is used as a powerful tool to assess rare and harmful effects of a treatment because it could allow increase clinical samples and improve productivity based on statistics, and we could achieve more stable estimates of effects in clinical practice. Preplanned MA of individual trials with deliberately introduced heterogeneity could help minimize different sources of bias and obtain a more generalizability result of harmful drug safety action from RCTs.^47^ However, some limitations should be aware in our MA. First, RCTs have strict inclusion and exclusion criteria and therefore the results of MA may not reflect the general patient population in everyday clinical practice. Secondly, MA based on published aggregated data tends to overestimate or underestimate treatment effects compared with individual patient data estimates.^48,49^ In addition, it obviated a more comprehensive evaluation, such as adjusting for baseline characteristic and other differences that existed between the different trials from which the data were pooled, although a previous study by Bennett demonstrated analogous results between the patient level and the study level of MA.^50^ Thirdly, the incidence of RA has a pattern of female prevalence worldwide, but because of insufficiency of original clinical data, the sex distribution data are unavailable in our study. Finally, trials which included in MA had clinically heterogeneous in terms of baseline characteristic, disease duration, and previous therapy.^26^ Therefore, inferences from our analysis of hypertension (which are derived from a mixed population of RA patients) on certain patient subsets should be caution.^51^

## CONCLUSION

In conclusion, our study suggests that the use of anti-TNF agent is associated with increased risk of developing hypertension. As these agents are increasingly used in the conventional therapy of patients with RA, physicians should be aware of this adverse effect and should monitor patients receiving anti-TNF agent continuously to offer early intervention and to obtain the balance between rheumatismal clinical benefit and adverse events. Further studies are still recommended to investigate this association.
